# Preschoolers’ and Mothers Dietary Practices and Compliance with the 24-h Movement Guidelines: Results of Oman’s National Nutrition Survey

**DOI:** 10.3390/ijerph18168867

**Published:** 2021-08-23

**Authors:** Salima Almaamary, Saleh Al Shammakhi, Ibtisam Alghamari, Jana Jabbour, Ayoub Al-Jawaldeh

**Affiliations:** 1 Nutrition Department, Ministry of Health, Muscat 393, Oman; dr.salima.almamary@gmail.com (S.A.); saleh9959@gmail.com (S.A.S.); ibtisam.alghammari@moh.gov.om (I.A.); 2 Nutrition Department, School of Health Sciences, Modern University of Business and Sciences, Beirut 113-7501, Lebanon; 3 Regional Office for the Eastern Mediterranean (EMRO), World Health Organization (WHO), Cairo 11371, Egypt; aljawaldeha@who.int

**Keywords:** childhood obesity, physical activity, media exposure, sleep hygiene, 24-h movement guidelines

## Abstract

Introduction: Little is known on the nutritional and lifestyle variables of preschool children in Oman. This study analyzed data of the 2017 Oman National Nutrition Survey to assess the prevalence and interrelationships between dietary and movement variables among preschool children, aged 2–5 years. Methods: Household visits of a nationally representative sample evaluated mothers and children’s dietary, sleep and physical activity practices; media exposure; and anthropometric and sociodemographic variables. Results: Dyads of mothers and pre-school children aged 2–5 years (*n* = 1771) were assessed. Childhood overweight/obesity was prevalent in 2% of the sample. Over 54% of children consumed sweetened items and/or French fries or chips at least once daily. Over 65% of children had fruits and/or vegetables once daily or less. Mothers had healthier dietary and movement habits compared to their offspring. Compliance with media exposure was the lowest among mothers and children. Multivariate regression revealed children’s increased sweet intake was the only significant predictor of excess weight in children. Conclusion: This study revealed a low prevalence of overweight/obesity among preschool children in Oman. Children had an acceptable compliance with sleeping recommendations, yet limited adherence to media exposure, activity, added sugar and fruits and vegetables guidelines.

## 1. Introduction

Childhood obesity is one of the most worrying public health problems of the century. Its burden stems from its immediate influence on the psychological, dental and skeletal wellbeing, its persistence in adolescence years, and its positive association with non-communicable diseases and mortality in adulthood [1,2,3,4,5]. Early identification of obesity plays a key role in controlling the disease at a time when lifestyle practices get established. Among preschoolers, the gradual rise of obesity raised the status of the challenge from important to urgent [6]. At the root cause of obesity in this population are poor dietary habits such as consumption of energy dense meals. Certain parents’ demographic variables such as education, Socio Economic Status (SES) and marital status influence the severity of this “disease” [7,8,9]. Additional predictors of childhood obesity include decreased physical activity (PA) and poor sleep hygiene, as well as duration and timing of media exposure [10,11,12,13]. Due to the importance of the latter variables, many societies have published 24-h movement guidelines identifying the importance of young children sleeping adequately, moving abundantly and limiting media exposure [14,15].

The Sultanate of Oman is a high-income country in the Eastern Mediterranean Region (EMR) and part of the Gulf Cooperation Council (GCC). The country has seen its health development index grow at exponential levels in the last 4 decades [16]. With the rise in wealth and commodity indices, obesity rates also increased in many childhood subgroups, reaching 5% among first graders (6–7 years), 15% among seven graders (12–13 years) and 16% among 10th graders (15–16 years) [17,18]. Children’s nutrition intake, parents’ education level, household income, nutrition and PA patterns were associated with obesity in middle childhood (5–10 year old) in Oman [17]. However, little is known on the obesity rates and nutritional and lifestyle factors of preschool children in the Sultanate. The Oman National Nutrition Survey (ONNS) was a national survey conducted in 2017 among children, up to 5 years old, and their mothers. This study analyzed data of the last ONNS to assess the prevalence and interrelationships between dietary and movement variables among preschool children, aged 2–5 years.

## 2. Materials and Methods

### 2.1. Sample Selection

The ONNS’ design was described in a previous publication [19]. Briefly, the ONNS adopted the World Health Organization (WHO)’s STEPS approach for sampling [20]. Household surveys were conducted between December 2016 and April 2017. Sampling data were taken from a previous STEPS survey, which included Omani and non-Omani residents. However, the ONNS included Omani residents only as it was meant to inform the strategic plans for the Ministry of Health. Samples were randomly chosen from each census block by governorate. A sample size of 375 households by governorate was identified as powerful to answer the primary research questions of the ONNS. An exception was granted for the Muscat governorate where all the households of Omani residents (*n* = 318) were selected. This was the case as Muscat hosts more foreigners than other governorates, and because the previous STEPS sampling data included Omani and non-Omani households, while the ONSS was only interested in Omani residents.

### 2.2. Data Collection

During the household visits, mothers who agreed to participate in the study provided consent for their participation as well as that of their children. Mothers were asked to answer questions related to household income, marital status, education level, working status and childcare facilities. The former variables as well as dietary and lifestyle practices were reported by mothers for themselves and their children. Dietary intake was assessed using a food frequency questionnaire evaluating intake in the seven days preceding the interview date. Questionnaires inquired about the frequency of consumption of fruits, vegetables, meat (processed vs. unprocessed), dairy, starchy foods, sweets (chocolates, sweets, candies, pastries, cakes or biscuits), French fries and chips, fast food meals, fruit juice (fresh vs. sweetened), soft drinks, energy drinks and coffee and tea (sweetened and unsweetened). Answers to these questions were reported as “Less than one time daily,” “one time daily,” “two times daily,” “three or more times daily” and “Don’t know.” For ease of presentation, during analysis, the research team recoded answers to “less than one time daily,” “one time daily” and “two or more times daily.” Children’s PA was evaluated through an assessment of weekly duration of walking, playing outdoors, participation in organized physical activities and attitudes towards PA. This time was then divided over seven and presented as a daily PA time in minutes. Mothers were asked to report the number of days the children walked to a destination (to get around in the neighborhood). The children media exposure was assessed using a questionnaire where mothers reported the children’s daily duration of exposure to TV, video, computer and mobile games, having a TV or computer in the child’s bedroom and the number of meals consumed while exposed to the media. Similarly, mothers were asked about their participation in organized physical activity (yes/no) and the type of organized exercise they were involved in. Habitual activity was assessed by inquiring if mothers walked or biked to do errands or work (yes/no). Intensity of physical activity was assessed for work activities that lasted for 10 or more minutes continuously. Intensity was classified as vigorous (for activities that cause significant increase in breathing rates) and moderate (for activities involving a slight increase in breathing rate). Duration (days, hours and minutes) was assessed for each of those activities. Sitting time was evaluated by asking the time spent sitting on weekdays and weekends. This time was averaged during analysis and presented as average daily time. Moreover, media exposure and frequency of intake of major food groups were evaluated using similar questions as those asked to children. Anthropometric measurements assessed children’s weight and length and the mothers’ weight, height, waist and hip circumferences [21]. Weight for height was calculated based on the WHO growth standard. Children who had a z scores < −2 were categorized as underweight, and those with a z score above 2 as overweight/obese, respectively [22]. A wealth index was calculated using the principal component analysis method, accounting for variables such as water, sanitation and possession of durable goods [14,15]. The wealth index was presented as quintiles with the first quintile reflecting the most prosperous category. Outcome assessors were trained on the questionnaires and measurements and piloted all the tools prior to data collection in the households.

### 2.3. Definitions

To assess the compliance of children with lifestyle recommendations for sleeping, media exposure and PA, these results were compared to age specific guidelines. Sleep was averaged for weekdays and weekends and followed the cut off points of the National Sleep Foundation [23]. Inadequate sleep duration was identified for 2–3-year-old and 3–5-year-old children who had a mean sleeping duration for <11 h and <10 h, respectively, and for children who had excessive sleep, i.e., 2–3-year-old and 4–5-year-old children who slept for >14 and 13 h, respectively. Children who had slept in between these brackets were deemed to have sufficient sleep. Mothers who had a mean sleeping duration of 7–9 h were identified to have sufficient sleep [23]. Media exposure and physical inactivity were assessed for mothers and offspring. Children who spent <1 h a day exposed to media [24] and adults who spent <2 h a day exposed to media not related to work [25] were compliant to the recommendations of media exposure. In the absence of a consensus on a cutoff point for sedentary time [26], we defined physical inactivity in mothers who had a sitting time ≥7 h/day. This figure was adopted from a prospective cohort study that evaluated the relationship between sitting behavior and all-cause mortality [27]. For children, the PA Guidelines for Americans 2018 recommend that preschool children should be active throughout the day [28]. We defined children as active if they played outdoors for the last 7 days for a minimum of 30 min a day on average.

### 2.4. Ethical Considerations

The ONNS’s followed the principles of the Declaration of Helsinki and received authorization for its conduct from the Research and Ethical Review and Approval Committee at Oman’s Ministry of Health. After explaining to them the goals, benefits and risks of the ONNS, mothers provided written informed consent to participate with their children in the survey.

### 2.5. Statistical Analysis

Categorical and continuous variables were presented using counts (percentages) and mean ± SD, respectively. Differences between variables were evaluated with the independent t-test for continuous variables, and the chi-square test for categorical variables. Missing variables were addressed using the WHO recommendations for dealing with missing data; cases with missing values were excluded for a determined variable [29]. To assess the predictors of excess weight among children (overweight/obese category), univariate and multivariate logistic regression were employed using the backward conditional method. Predictors with *p*-values < 0.15 at the univariate level were incorporated in the multivariate model [30]. Data analysis was conducted on IBM-SPSS (version 25.0; IBM Corp. Released 2017. IBM SPSS Statistics for Windows, IBM Corp., Armonk, NY, USA).

## 3. Results

Dyads of mothers and pre-school children aged 2–5 years (*n* = 1771) were included in the analysis. Children had similar representation of the different age and sex categories (Table 1). Most children (>85%) were cared for at home with parents and relatives rather than in preschool facilities. As for the mothers, their mean age was 33 years and most of them received secondary education, did not work outside their homes and had a mean BMI of 29.4 Kg/m^2^ (Table 1).

**Figure 1:**
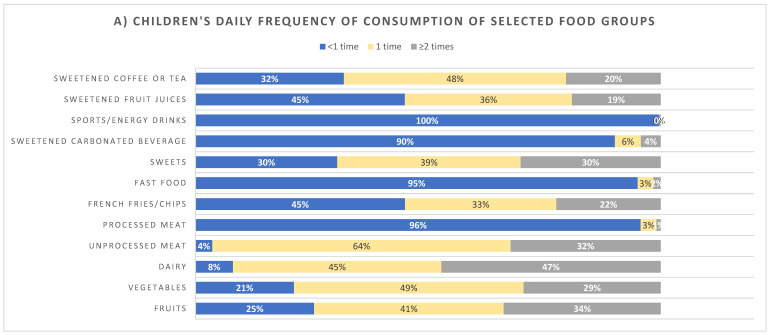

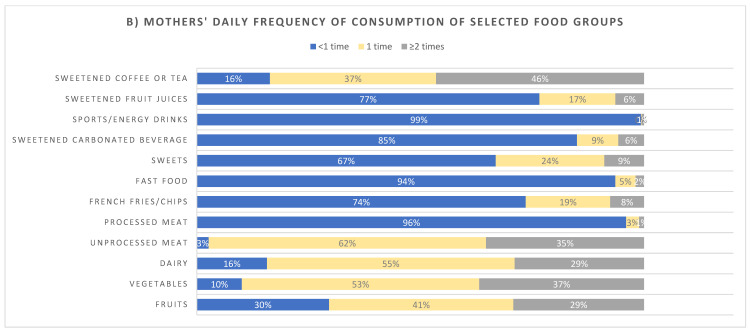
Daily frequency of consumption of selected food groups of (**A**) children (top panel) and (**B**) mothers (bottom panel).

Figure 1 presents the frequency of consumption of selected food groups of children (Figure 1A) and mothers (Figure 1B). On the one hand, over 54% of children consumed sweets, sweetened coffee/tea, sweetened juices or French fries or chips at least once daily. Moreover, over 65% of children consumed fruits and vegetables once daily or less. Mothers, on the other hand, had healthier dietary trends compared to their offspring, with 9% only consuming sweets ≥twice daily compared to 30% of children (*p* = 0.082), and 27% consuming French fries/chips at least once daily compared to 55% of children (*p* = 0.44).

Table 2 summarizes the PA and media exposure of children and mothers. Around 41% of children walked to a destination most days of the week. On average, children played outdoors for 30 min or more 5 days a week. A small percentage of preschoolers (13%) participated in structured PA, and the mean daily PA duration of children was 22 min for all activities combined. Around one third of children were exposed to media outlets while eating on a daily basis and had a TV or a computer in their bedroom (Table 2). As for mothers, few walked on a daily basis for errands and about one quarter participated in structured PA. Around 10% of mothers had work errands that required vigorous or moderate PA. When mothers’ work involved vigorous or moderate PA, its duration ranged between 17 and 23 h per week. Daily media exposure during meals was less frequent among mothers with only 5% of women consuming their meals while watching TV (Table 2).

**Figure 2:**
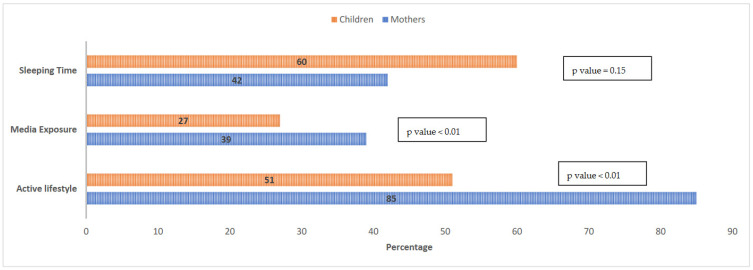
Compliance with Sleeping, Media Exposure and PA guidelines among Mothers and Children. *p*-values represent differences between mothers and children calculated using the independent t test.

Figure 2 displays the frequency of children and mothers compliant to lifestyle patterns. About 60% of children and 42% mothers were compliant to sleeping recommendations with no significant differences between groups. Mothers had better compliance with media exposure and activity recommendations compared to their children (*p* < 0.01). Compliance with media exposure was the lowest in both groups, with 27% and 39% of children and mothers, respectively, found to be compliant with the relevant guideline (Figure 2). Univariate and multivariate logistic regression of children’s overweight/obesity is displayed in Table 3. Even though mother’s BMI, wealth quintile and governorate were significant predictors at the univariate level, they lost significance at the multivariate level. The only significant predictor of overweight/obesity at the multivariate level was the intake of sweets, with a consumption of ≥1 sweet serving daily being associated with excess weight (adjusted odds ratio: 2.8; *p*-value = 0.029).

## 4. Discussion

This study provided an overview of toddlers and preschool children’s dietary and lifestyle patterns in Oman from a nationally representative sample. Results revealed preschool children had low rates of overweight and obesity and acceptable compliance with sleeping recommendations, yet limited adherence to media exposure, activity, added sugar and fruits and vegetables guidelines. Mothers had better compliance with activity, media exposure recommendations and a reduced added sugar intake compared to their children.

This national survey revealed that only around 2% of Omani preschool children are overweight and/or obese. These figures are markedly lower than those documented in neighboring countries of the EMR such as Egypt, Lebanon, the Kingdom of Saudi Arabia (KSA) and Kuwait, where figures ranged between 6% and 37% [7,8,31,32] and those of American and European countries where prevalence has reached 30% [33,34,35]. Omani children had low consumption of fast-food meals, soft drinks and processed meat. They nevertheless had an increased intake of sweets, sweetened beverages and French fries/chips. Mothers tended to have healthier diets than their children with a reduced intake of sweets, sweetened beverages and chips and an increased intake of vegetables.

Omani children had an acceptable compliance with sleeping guidelines with around 60% achieving the National Sleep Foundation recommendations for their age [23]. Compliance to media exposure guidelines was low, with more than 70% of children being exposed to media outlets for one hour or more daily. This finding may be associated with the fact that the majority of assessed children did not attend preschool educational facilities. Most children were attended for at home by their parents and relatives. This finding is likely associated with the fact that 83% of mothers did not work outside their homes. In a high-income country such as Oman, there may be less financial pressure on both parents to work. These findings echo those found in KSA, another high income country in the GCC, where 63% of 3–5 year old children did not attend schools [12]. Even though univariate regression in our study did not show a relationship between child obesity and mother’s employment status, the literature on the subject suggests a possible relationship between these two variables. Studies from the EMR found that mother’s unemployment had a negative impact on children’s sleep score in KSA and was associated with increased consumption of fast-food meals among middle age children in Oman [12,36]. While a systematic review from low-income countries found no association between maternal employment and obesity, analyses from European countries concluded that mother employment predicted unhealthy dietary, lifestyle patterns and increased BMI among young children [37,38,39]. Of relevance, results of the latter analyses were more pronounced among single mothers and parents who had long working hours [38,39]. The controversy in this topic identifies the need for further assessment of the relationship of parents’ employment status, working hours and childhood obesogenic environments.

Omani children had lower adherence with PA and sleep guidelines and better compliance with screen time recommendations than their Australian and Finnish peers [40,41]. A cross sectional study assessing sleep time and quality of Middle Eastern children had found that young children living in the Middle East region had worse sleep quality and duration compared to their peers residing in predominantly Asian and Caucasian countries [42]. Compared to other countries within the EMR, Omani children had better adherence with PA and screen time recommendations than Saudi children [12,43,44], yet worse adherence with sleep and media exposure than their Emirati peers [45].

Multivariate regression in our study revealed that increased intake of sweets was associated with increased odds of obesity. These results echo findings from Lebanon that showed that a “fast food & sweets” dietary pattern increased the risk of obesity compared to a Lebanese Mediterranean pattern [46]. A study among middle age children in Oman had shown a relationship between children’s BMI, maternal education and BMI [36]. However, the latter variables did not predict increased BMI in our logistic regression analysis. This may suggest that such determinants have a more pronounced impact in middle compared to early childhood.

### Strengths & Limitations

This study had several strengths and limitations. On the one hand, it analyzed a large and nationally representative sample. Moreover, it addressed a literature gap on obesity and lifestyle patterns among preschool children, by analyzing data on some of the confounding variables of obesity among both mothers and children. These findings are of relevance in Oman in view of the scarcity of data in this age group. Limitations of the study included the use of non-validated questionnaires to assess dietary patterns. Even though the dietary questionnaire was piloted on the sample prior to its administration, it was not validated on this population. Moreover, PA data were reported rather than measured, which is associated with a recall bias. The latter limitation is inherent to such national surveys due to the cost-burden and difficulty of tracking such a large and dispersed sample. Finally, questionnaires focused on mothers’ education and lifestyle patterns only. An assessment of fathers’ dietary and movement patterns would have further enriched the evaluation.

## 5. Conclusions

This is the first national study to our knowledge that describes dietary and lifestyle patterns among preschool children and mothers in Oman and which associate them with obesogenic outcomes. The findings revealed that the majority of children stayed at home and were cared for by non-working mothers. Prevalence of overweight/obesity was low among Omani children compared to regional and worldwide figures. Children had an acceptable compliance with sleeping recommendations, yet limited adherence to media exposure, activity, added sugar and fruits and vegetables guidelines. Increased sugar intake for children was the only predictor of overweight/obesity. Given the rates of increased obesity in middle childhood in Oman, future interventions should address ways to optimize compliance of preschool children with media exposure, physical activity and dietary guidelines by addressing both children and parents [47].

## Figures and Tables

**Table 1:** Children and mothers’ socio demographic characteristics.

Characteristic		Result (*n* = 1711)
Age category, *n*(%)	2–3 years	637 (36)
	3–4 years	609 (34)
	4–5 years	525 (30)
Child sex, *n*(%)	Male	904 (51)
	Female	867 (49)
Child care facilities, *n*(%)	At home with parents	902 (51)
	At home with relatives	619 (35)
	Day Care	100 (5.7)
	Preschool	115 (6.5)
	Other	7 (0.4)
Children weight to height category, *n*(%)	Underweight	207 (12)
	Normal Weight	1525 (86)
	Overweight/Obese	39 (2.0)
Mother’s Body Mass Index (Kg/m^2^), mean ± SD		29 ± 6.6
Worked outside their homes, *n*(%)		300 (17)
Wealth index, *n*(%)	Poorest	281 (16)
	Poor	369 (21)
	Middle	350 (20)
	Wealthy	372 (21)
	Wealthiest	372 (21)
Governorate, *n*(%)	Muscat	70 (3.4)
	Dhofor	207 (12)
	Al Dhakhlya	190 (11)
	Al-Sharqyah North	151 (8.5)
	Al-Sharqyah South	150 (8.5)
	Al-Batinah North	174 (9.8)
	Al-Batinah South	164 (9.3)
	Al-Dhahairah	171 (9.7)
	Al-Buraimy	168 (9.5)
	Musandam	149 (8.4)
	Al-Wusta	177 (10)

**Table 2:** Lifestyle characteristics of children and mothers.

Characteristic		Result (*n* = 1711)
Children		
Days/week child walked to go to a destination, *n*(%)	0–1	651 (38)
	2–3	354 (21)
	4–7	354 (41)
Days per week child played outdoors for ≥30 min, mean ± SD		4.7 ± 2.7
Participation in structured PA, *n*(%)		237 (13)
Estimated physical activity (minutes/day), mean ± SD		22 ± 15
Attitude towards PA, *n*(%)	Does not like it	15 (0.9)
	Likes it	927 (53)
	Likes it a lot	804 (46)
Media exposure during meals, *n*(%)	Rarely	1152 (65)
	1–2 meals/day	536 (31)
	≥3 meals/day	63 (3.6)
Having a TV or computer in the bedroom, *n*(%)		506 (29)
Mothers		
Habitual activity (walk or bike to do errands), *n*(%)		13 (4.5)
Participation in organized PA, *n*(%)		438 (26)
Work involves vigorous PA, *n*(%)		8 (2.5)
Weekly hours of vigorous PA, mean ± SD		17 ± 14.7
Work involves moderate PA, *n*(%)		141 (8.0)
Weekly hours of moderate PA, mean ± SD		23 ± 0.10
Media exposure during meals, *n*(%)	Rarely	1581 (95)
	1–2 meals/day	323 (4.3)
	≥3 meals/day	27 (1.6)
Having a TV or computer in the bedroom, *n*(%)		659 (40)

PA: Physical activity.

**Table 3:** Logistic Regression of children being in the overweight or obese category.

Variable	Univariate Analysis			Multivariate Analysis		
	OR	95% CI	*p* Value	AOR	95% CI	*p* Value
Child’s Female sex	0.59	0.31–1.1	0.117			
Days child played outside for ≥30 min	0.99	0.87–1.1	0.863			
Child’s compliance with sleeping guidelines	1.0	0.54–2.0	0.894			
Child’s compliance with media exposure guidelines	0.9	0.46–1.7	0.755			
Child’s compliance with physical activity guidelines	0.9	0.49–1.8	0.821			
Child’s intake of sweets >once daily	1.9	0.84–4.4	0.120	2.8	1.1–7.1	0.029
Child’s intake of vegetables > once daily	0.72	0.37–1.4	0.341			
Mother’s age (years)	1.0	0.99–1.1	0.219			
Mothers’ BMI (Kg/m^2^)	0.95	0.91–0.99	0.019	0.97	0.92–1.0	0.288
Mother’s education Level			0.770			
Less than primary (referent)	1	-	-			
Primary	N/A					
Secondary	0.77	0.18–3.3	0.729			
Tertiary	0.55	0.12–2.5	0.440			
Mother working outside her home	1.7	0.54–5.7	0.361			
Wealth Quintile			0.043			0.691
Poorest (referent)	1	-	-	1	-	-
Poor	2.2	0.52–9.2	0.289	0.941	0.15–5.8	0.948
Middle	1.2	0.36–4.4	0.730	0.719	0.11–4.9	0.736
Wealthy	0.48	0.17–1.4	0.162	0.455	0.07–3	0.409
Wealthiest	0.47	0.17–1.3	0.157	0.390	0.06–2.5	0.318
Governorate			<0.01			0.053
Muscat (referent)	1	-	-	1	-	-
Dhofor	1.0	0.33–3.3	0.952	0.52	0.11–2.5	0.413
Al Dhakhlya	14.5	1.6–133	0.018	8.4	0.74–96	0.086
Al-Sharqyah North	5.5	0.98–31	0.053	3.5	0.47–26	0.225
Al-Sharqyah South	5.6	0.99–31	0.051	2.8	0.38–21	0.316
Al-Batinah North	3.1	0.75–13	0.119	1.7	0.3–9.6	0.557
Al-Batinah South	11.5	1.3–105	0.031	6.8	0.59–77	0.123
Al-Dhahairah	N/A			N/A		0.996
Al-Buraimy	2.0	0.55–7.5	0.289	1.4	0.25–7.4	0.712
Musandam	N/A			N/A		0.996
Al-Wusta	3.3	0.79–14	0.104	1.4	0.24–8.2	0.700

Variables that had a *p*-value < 0.15 in the univariate regression are presented in the multivariate analysis.

## Data Availability

3rd Party Data Restrictions apply to the availability of these data. Data was obtained from the Ministry of Health, from the authors Salima Al Mamary and Saleh Al Shammakhi.
